# Potential drug-drug interaction of olverembatinib (HQP1351) using physiologically based pharmacokinetic models

**DOI:** 10.3389/fphar.2022.1065130

**Published:** 2022-12-13

**Authors:** Zhiheng Yu, Zihan Lei, Xueting Yao, Hengbang Wang, Miao Zhang, Zhe Hou, Yafen Li, Yangyu Zhao, Haiyan Li, Dongyang Liu, Yifan Zhai

**Affiliations:** ^1^ Drug Clinical Trial Center, Department of Obstetrics and Gynecology, Peking University Third Hospital, Beijing, China; ^2^ Center of Drug Metabolism and Pharmacokinetics, School of Basic Medicine and Clinical Pharmacy, China Pharmaceutical University, Nanjing, China; ^3^ Drug Clinical Trial Center, Peking University Third Hospital, Beijing, China; ^4^ Drug Clinical Trial Center, Institute of Medical Innovation and Research, Peking University Third Hospital, Beijing, China; ^5^ Guangzhou Healthquest Pharma Co., Ltd, Guangzhou, China; ^6^ Department of Obstetrics and Gynecology, Peking University Third Hospital, Beijing, China; ^7^ Drug Clinical Trial Center, Department of Cardiology and Institute of Vascular Medicine, Institute of Medical Innovation and Research, Peking University Third Hospital, Beijing, China

**Keywords:** chronic myeloid leukemia, cytochrome P450, drug-drug interactions, modeling, physiologically based pharmacokinetics, tyrosine kinase inhibitor

## Abstract

Olverembatinib (HQP1351) is a third-generation BCR-ABL tyrosine kinase inhibitor for the treatment of chronic myeloid leukemia (CML) (including T315I-mutant disease), exhibits drug-drug interaction (DDI) potential through cytochrome P450 (CYP) enzymes CYP3A4, CYP2C9, CYP2C19, CYP1A2, and CYP2B6. A physiologically-based pharmacokinetic (PBPK) model was constructed based on physicochemical and *in vitro* parameters, as well as clinical data to predict 1) potential DDIs between olverembatinib and CYP3A4 and CYP2C9 inhibitors or inducers 2), effects of olverembatinib on the exposure of CYP1A2, CYP2B6, CYP2C9, CYP2C19, and CYP3A4 substrates, and 3) pharmacokinetics in patients with liver function injury. The PBPK model successfully described observed plasma concentrations of olverembatinib from healthy subjects and patients with CML after a single administration, and predicted olverembatinib exposure increases when co-administered with itraconazole (strong CYP3A4 inhibitor) and decreases with rifampicin (strong CYP3A4 inducer), which were validated by observed data. The predicted results suggest that 1) strong, moderate, and mild CYP3A4 inhibitors (which have some overlap with CYP2C9 inhibitors) may increase olverembatinib exposure by approximately 2.39-, 1.80- to 2.39-, and 1.08-fold, respectively; strong, and moderate CYP3A4 inducers may decrease olverembatinib exposure by approximately 0.29-, and 0.35- to 0.56-fold, respectively 2); olverembatinib, as a “perpetrator,” would have no or limited impact on CYP1A2, CYP2B6, CYP2C9, CYP2C19, and CYP3A4 enzyme activity 3); systemic exposure of olverembatinib in liver function injury with Child-Pugh A, B, C may increase by 1.22-, 1.79-, and 2.13-fold, respectively. These simulations inform DDI risk for olverembatinib as either a “victim” or “perpetrator”.

## 1 Introduction

Olverembatinib (HQP1351) is a third-generation BCR-ABL1 tyrosine kinase inhibitor (TKI) (Ren et al., 2013). In 2020, the China National Medical Products Administration (NMPA) Center for New Drug Evaluation designated olverembatinib as a potential breakthrough therapy for treatment of patients with chronic myeloid leukemia (CML) that is resistant and/or intolerant to first- and second-generation TKI therapies (Center for Drug Evaluation, 1211). Shortly afterward, the NMPA approved conditional marketing authorization for olverembatinib (breakthrough therapy designation, 1211) at a dose of 40 mg orally every other day (QOD). Olverembatinib is currently approved by the US Food and Drug Administration as an orphan drug to treat CML, acute lymphoblastic leukemia, and acute myeloid leukemia (Orphan Drug Designations and Approvals).

Olverembatinib binds tightly to the ATP-binding sites of native BCR-ABL1 and multiple BCR-ABL1 mutants, including the most refractory (“gatekeeper”) mutant T315I, and potently suppresses proliferation of leukemia cells expressing BCR-ABL1 (Ren et al., 2013). Compared with third-generation TKI inhibitor ponatinib, olverembatinib exhibited equivalent or more potent antiproliferative activity in both imatinib-resistant and imatinib-sensitive gastrointestinal stromal tumor cell lines (Liu et al., 2019). Clinical trials of olverembatinib have demonstrated preliminary safety and effectiveness in patients with T315I-mutated CML in the chronic phase (CML-CP) and accelerated phase (CML-AP). When administered at 40 mg QOD for 28 consecutive days per cycle over 24 months, olverembatinib elicited a major cytogenetic response (MCyR) in 79.3% patients with CML-CP (n = 121 without MCyR at baseline) and a major hematologic response (MaHR) in 78.4% patients with CML-AP (n = 37) without MaHR at baseline (Jiang et al., 2022).

Clinical pharmacokinetic data (data on file) have shown that olverembatinib is rapidly absorbed and slowly eliminated after oral administration in fasting and fed patients with CML. The median time to reach peak plasma concentration (T_max_) for olverembatinib is 6 h, with a mean apparent terminal elimination half-life (t_1/2_) of 32.7 h. There is no significant food effect; the maximum plasma concentration (C_max_) and area under the plasma concentration-time curve from time 0 to infinity (AUC_0-∞_) ratio of fed to fasting is 1.28 and 1.17, respectively. After administration of a single radiolabeled dose of olverembatinib (30 mg) in healthy subjects, 87.68% of the dose was recovered in feces within 216 h, and 1.57% was recovered in urine within 96 h. About 23.95% of the cleared drug was unchanged in feces and 0.02% eliminated as the parent drug in urine.

Cytochrome P450 (CYP3A4 and CYP2C9)-mediated metabolism is a major clearance pathway. In the parlance of drug-drug interaction (DDI) studies, the “perpetrator” is the medication that affects the pharmacokinetics of another agent, whereas the “victim” is the medication whose pharmacokinetics are affected by the perpetrator. An olverembatinib clinical DDI study (data on file) showed that coadministration with itraconazole, a substrate for and inhibitor of CYP3A4, increased olverembatinib C_max_ by 1.74-fold and increased area under the concentration-time curve (AUC) by 2.63-fold. On the other hand, coadministration with CYP3A4 inducer rifampicin decreased the olverembatinib maximum concentration (C_max_) and area under the concentration-time curve (AUC) by 0.36-fold and 0.24-fold, respectively. However, the potential for DDI between olverembatinib and other CYP3A4 inhibitors or inducers was not evaluated in DDI clinical trials. The DDI risk of olverembatinib in combine with CYP2C9 inhibitors or inducers was also not evaluated. Moreover, olverembatinib inhibits CYP2C9 and CYP2C19, and induces CYP1A2, CYP2B6, and CYP2C9, *in vitro*. The effect of olverembatinib as a perpetrator was also not evaluated in previous clinical DDI studies.

Increasingly, physiologically-based pharmacokinetic (PBPK) modeling applications have been used to facilitate the design of DDI studies, evaluate DDI potential *in lieu* of dedicated clinical studies in new drug applications (Zhao et al., 2011; Jones et al., 2015; Shebley et al., 2018). PBPK models that have been validated with clinical data have been increasingly used to predict the DDI potential for untested scenarios. To fully assess DDI risk, evaluate pharmacokinetic changes in patients with liver function injury, the primary objective of this study was to develop and verify a PBPK model for a comprehensive assessment of its clinical DDI and pharmacokinetics profiles in patients with liver function injury.

## 2 Methods

### 2.1 Modeling strategy

**FIGURE 1:**
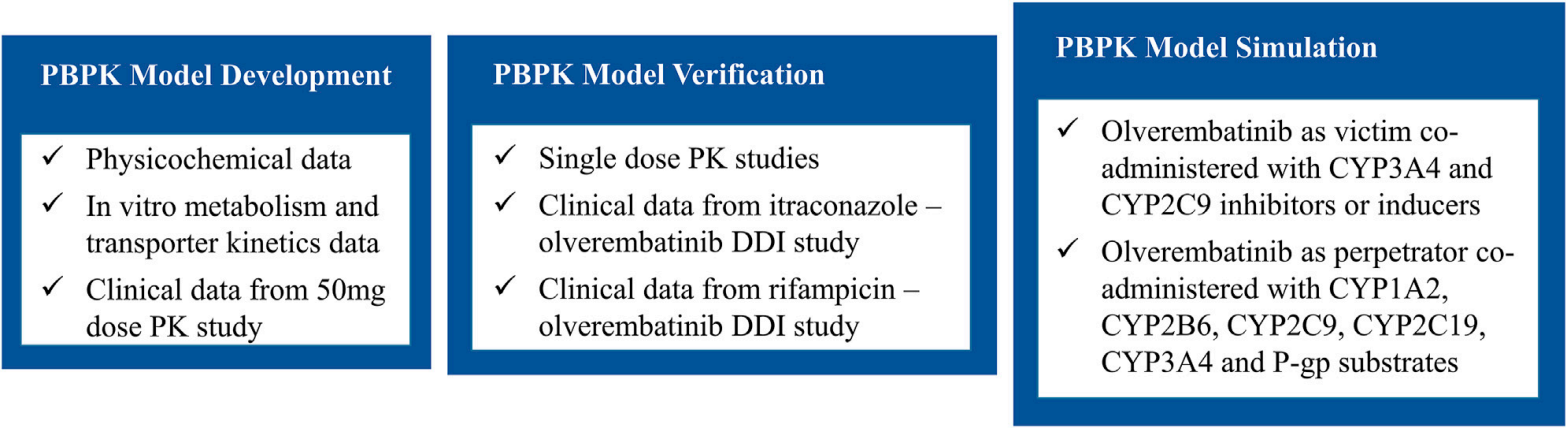
The overall model development, verification process, and simulation flow chart.

We formulated a overall strategy for qualification of the olverembatinib model and prediction of DDI (Figure 1). An olverembatinib PBPK model was constructed to simulate the plasma concentration-time profiles of olverembatinib after single-dose administration in healthy volunteers and patients with CML. To evaluate the ability of the model to predict DDI associated with olverembatinib, we conducted clinical DDI studies with strong CYP3A inhibitor and antifungal agent itraconazole and strong CYP3A inducer rifampicin to further verify the base PBPK model. Prespecified acceptance criteria (0.5–2.0-fold of observed values) were used to guide model construction and validation.

### 2.2 Clinical data

All clinical pharmacokinetic data included in the modeling were obtained from three clinical studies (Supplementary Table S1). These studies were conducted in accordance with ethical standards of the institutional and/or national research committee, as well as the Declaration of Helsinki. Institutional review boards or equivalent ethics committees approved the study protocols, and all study participants provided written informed consent before any study-related procedures.

In the first study, on food effects, 12 Chinese patients with CML were randomly divided into two groups (A and B) and the experiments divided into two periods. In period 1, subjects in group A received 30 mg of oral olverembatinib under fasting conditions, while subjects in group B received the same oral dose 30 min after a high-fat meal. After a seven- day washout period, subjects in both groups took the drug interchangeably in period 2 (NCT03882281).

The second study was a single-center, open-label, single-dose phase 1 trial to investigate the absorption, metabolism, and excretion of [^14^C]olverembatinib after a single oral 30 mg (100 µCi) dose in six healthy Chinese male subjects (NCT04126707). The third study was a phase 1, two-part trial designed to evaluate the interaction of olverembatinib and CYP3A4 perpetrators in healthy American subjects (n = 32). For uninhibited periods, subjects received one oral 20 mg dose of olverembatinib on Day 1. After a 3-day washout period, they were pretreated with 200 mg of oral itraconazole once daily for 4 days to inhibit CYP3A4 (Days 5–8). On Day 9, subjects received single oral 20-mg doses of olverembatinib and itraconazole, or itraconazole alone on Days 10 through12. For uninduced periods, subjects received one oral 40 mg dose of olverembatinib on Day 1. After a 3-day washout, subjects were pretreated with 600 mg of oral rifampicin once daily for 7 days to induce CYP3A4 (Days 5–11). On Day 12, they received single oral doses of olverembatinib at 40 mg and rifampicin at 600 mg, as well as rifampicin alone on Days 13 through15. Blood samples were collected immediately before and after drug administration at 0.5, 1, 2, 4, 6, 8, 12, 24, 48, 72, and 96 h.

### 2.3 Model construction

A minimal PBPK model with a first-order absorption model was used to construct the olverembatinib PBPK model, including model input parameters (Table 1). The first-order absorption model has different absorption rate constants (k_a_) and fraction available from dosage form (f_a_) to simulate absorption processes in fasting and fed states. The effective permeability of olverembatinib in the human jejunum was based on built-in predictive *in silico* tools (Sun et al., 2002) within the simulator and *in vitro* Caco-2 cell permeability data with correction by reference drugs (β-adrenoceptor blockers atenolol and propranolol). Caco-2 (human colorectal adenocarcinoma) cells are used to model the intestinal epithelial barrier. The Simcyp (Version 19.0, Certara Inc., Sheffield, UK) minimal PBPK, which treats all organs other than the intestine and liver as a single compartment (Rowland Yeo et al., 2010), was selected, along with a single adjusting compartment (SAC) distribution model. The SAC is a nonphysiologic compartment that permits adjustment to the drug concentration profile in the systemic compartment (Ke et al., 2016).

**TABLE 1:** Model development and model parameters.

Parameter	Value	Source
MW (g/mol)	532.56	Internal data
f_u,p_	0.0005	Experimental data^a^
Log P_o:w_	3.6	Experimental data^a^
pKa 1	2.90	Experimental data^a^
pKa 2	10.89	Experimental data^a^
B/P ratio	1.29	Experimental data^a^
Absorption model		
k_a_ (1/h)	0.136 (Fed)	Optimized based on observed data
	0.213 (Fasting)	
f_a_	0.8 (Fed)	Optimized based on observed data
	0.574 (Fasting)	
P_app_ (10^–6^ cm/s) (HQP1351)	0.08	Experimental data^a^
P_app_ (10^–6^ cm/s) (Atenolol)	0.38	Experimental data^a^
P_app_ (10^–6^ cm/s) (Propranolol)	5.68	Experimental data^a^
Distribution model		
V_sac_ (L/kg)	38.02	Optimized based on observed data
SAC k_in_ (1/h)	0.0471	Optimized based on observed data
SAC k_out_ (1/h)	0.0177	Optimized based on observed data
K_p_ Scalar	6.01	Optimized based on observed data
Elimination model		
CL_int_ _CYP2C9_ (µL/min/pmol CYP)	0.022	Experimental data^a^
CL_int_ _CYP3A4_ (µL/min/pmol CYP)	0.2	Experimental data^a^
CL_int_ _HLM_ (µL/min/mg protein)	5.2559	Optimized based on liver microsomal enzyme phenotype study and human recombinant CYP enzyme study
f_u,mic_/f_u,inc_	0.0005	Assumed equal to fu,p
ISEF _CYP2C9_	1.2938	Optimized based on fm_CYP2C9_ with liver microsomal enzyme phenotype study and human recombinant CYP2C9 study
ISEF _CYP3A4_	0.7761	Optimized based on fm_CYP3A4_ with liver microsomal enzyme phenotype study and human recombinant CYP3A4 study
CLr (L/h)	0.132	Based on phase 1 dose escalation clinical study
Drug interaction		
Ki _CYP2C9_ (µM)	1.135	Calculated with IC_50,_ substrate concentration^a^, and substrate K_m_ ^b^
Ki _CYP2C19_ (µM)	1.924	Calculated with IC_50,_ substrate concentration^a^, and substrate K_m_ ^c^
Ind_max CYP1A2_	6.26	Experimental data^a^
Ind_max CYP2B6_	17.900	Experimental data^a^
Ind_max CYP2C9_	17.150	Experimental data^a^
IndC_50 CYP1A2_ (µM)	0.15	Experimental data^a^
IndC_50 CYP2B6_ (µM)	0.559	Experimental data^a^
IndC_50 CYP2C9_ (µM)	0.454	Experimental data^a^
Ki _P-gp_ (µM)	2.23	Calculated with IC_50,_ substrate concentration^a^ and substrate Km^d^

^a^ Internal data (fu, B/P ratio, CLintCYP, IndmaxCYP, and IndC50CYP, are summarized in Supplementary Tables S4-S7).

^b^ Data were derived from Lee MY, Borgiani P, Johansson I, Oteri F, Mkrtchian S, Falconi M, et al. (Lee et al., 2014).

^c^ Data were derived from Yang S, Qiu Z, Zhang Q, Chen J, Chen X (Yang et al., 2014).

^d^ Data were derived from Troutman MD, Thakker DR. (Troutman and Thakker, 2003) MW, molecular weight; fu,p, fraction unbound in plasma; logP, log of the octanol-water partition coefficient for the neutral compound; pKa, acid dissociation constant; B/P, blood/plasma partition ratio; ka, first-order absorption rate constant; fa, fraction available from dosage form; Vsac, volume of the single adjusting compartment; SAC, single adjusting compartment; kp scalar, scalar applied to all predicted tissue kp values; CLint, intrinsic clearance; CYP, cytochrome P450; HLM, human liver microsome; fu,mic, microsomal protein binding; fmCYP2C9, fraction of drug metabolized by CYP2C9; fmCYP3A4, fraction of drug metabolized by CYP3A4; CLR, renal clearance; Ki, enzyme or transporter inhibition constant (concentration of inhibitor associated with half maximal inhibition); IC_50_, half-maximal inhibitory concentration; Indmax, calibrated maximal fold induction over vehicle (1 = no induction); IndC50, calibrated inducer concentration that supports half-maximal induction (μM).

Final values of the apparent volume of SAC (V_sac_), as well as the rate constants from systemic compartment to SAC (*K*_*in*_) and from SAC compartment to the systemic compartment (*K*_*out*_), were optimized based on observed pharmacokinetic data. The volume of distribution at steady state (V_ss_) was predicted from the steady-state tissue: plasma partition coefficient (*K*_*p*_) calculated using mechanistic tissue distribution equations (Rodgers et al., 2005; Rodgers and Rowland, 2006). Intrinsic clearance values of 0.200 μL/min/pmol and 0.022 μL/min/pmol were assigned for CYP3A4 and CYP2C9, respectively, based on an *in vitro* human recombinant CYP isoenzyme study. Intersystem extrapolation factors for CYP3A4 and CYP2C9 were optimized based on clinical metabolism and excretion data in a mass balance study (NCT04126707). The mean value of renal clearance was confirmed by a phase 1 dose escalation study in 12 Chinese patients who received 50 mg of oral olverembatinib on Day 1.

Olverembatinib exhibits CYP2C9 and CYP2C19 inhibition and CYP1A2, CYP2B6 and CYP2C9 induction *in vitro*. The concentration of olverembatinib that supports half-maximum inhibition (*K*_*i*_) was calculated using Eq. 1.

$$K_{i}=\frac{IC50}{1+\left[S\right]/K_{m}}$$ (1)

Where IC_50_ signifies the half-maximum inhibitory concentration; S, the concentration of P450 marker substrate; and *K*_*m*_, the Michaelis constant within the Michaelis-Menten equation, which is in turn used to characterize substrate-enzyme binding kinetics.

As a CYP2C9 probe substrate, the nonsteroidal anti-inflammatory drug diclofenac (2.5 µM) was incubated with human liver microsomes, nicotinamide adenine dinucleotide phosphate, and olverembatinib to obtain the IC_50_. The Michaelis constant (*K*_*m*_) of CYP2C9 incubated with diclofenac was obtained from reported data (Lee et al., 2014). Methods used to calculate the K_i_ of CYP2C19 and CYP2C9 were similar. The maximum fold induction (Ind_max_) and half-maximum fold induction concentration (IndC_50_) values of olverembatinib on CYP1A2, CYP2B6, and CYP2C9 were calculated by plotting mRNA fold induction *versus* olverembatinib concentration after *in vitro* incubation with human hepatocytes.

### 2.4 Model validation

The olverembatinib PBPK model was constructed and validated using Simcyp. Default virtual population models of healthy White and Chinese volunteers were used, except for demographic data noted in each clinical study. For model construction and validation, the virtual trials used in each simulation were based on corresponding doses used in each of the clinical studies (Supplementary Table S1). The constructed PBPK model was validated by comparing the predicted model and observed pharmacokinetic parameters and/or plasma concentration profiles. The PBPK model predictive performance was preliminarily evaluated by overlaying the observed concentration-time profile with the model-predicted profile and 90% predictive interval. The quantitative assessment was conducted by pharmacokinetic parameters (C_max_ and AUC), as represented by the ratio of the predicted to the observed value. The model performance success criterion was acceptable if it fell within the 0.5- to 2.0-fold range.

### 2.5 Model hypothesis

A food effect study (NCT03882281) was conducted in Chinese patients with CML, for whom there was no CML population library in Simcyp. As a result, a “Sim-Chinese healthy volunteers” simulated population was used for model verification under the assumption of equivalent pharmacokinetics in patients with CML and healthy volunteers.

### 2.6 Model simulation

After validation, the PBPK model was used to simulate untested clinical DDI scenarios for olverembatinib as either a victim or perpetrator (Zhang et al., 2022). As a victim, olverembatinib pharmacokinetics were simulated after coadministration with CYP3A4 and CYP2C9 perpetrators according to a prespecified dose scheme (Table 2). Substrate pharmacokinetics of CYP2C9, CYP2C19, CYP1A2, CYP2B6, and P-glycoprotein (P-gp) were simulated after coadministration with olverembatinib (as a perpetrator), also according to a protocol-based dose scheme (Table 2).

**TABLE 2:** Simulated C_max_ and AUC ratios of olverembatinib (HQP1351) as victim in the presence and absence of CYP modulators and simulated C_max_ and AUC of CYP substrates when olverembatinib as CYP modulators.

Inhibitor/inducer	Dose	Treatments days	HQP1351 dose	HQP1351 treatments	C_max___inh_ (ng/ml)	AUC_96___inh_ (h•ug/mL)	C_max_ ratio	AUC ratio
CYP3A4								
Itraconazole (strong inhibitor)	200 mg QD	8	40 mg	Coadministration on day 5	28.8	1.05	1.64	2.39
Verapamil (moderate inhibitor)	80 mg TID	8	40 mg	Coadministration on day 5	27.7	0.99	1.55	2.25
Fluconazole (moderate inhibitor)	200 mg QD	10	40 mg	Coadministration on day 7	24.9	0.79	1.42	1.80
Erythrocin (moderate inhibitor)	500 mg Q6h	8	40 mg	Coadministration on day 5	28.4	1.05	1.60	2.39
Fluvoxamine (mild inhibitor)	50 (36.65) mg QD^d^	10	40 mg	Coadministration on day 7	18.4	0.48	1.05	1.08
Cimetidine (mild inhibitor)	400 mg BID	10	40 mg	Coadministration on day 7	18.4	0.47	1.05	1.08
Rifampicin (strong inducer)^a^	600 mg QD	11	40 mg	Coadministration on day 8	7.56	0.13	0.43	0.29
Efavirenz (moderate inducer)	600 mg QD	11	40 mg	Coadministration on day 8	8.59	0.15	0.48	0.35
Phenobarbital (moderate inducer)^b^	100 mg QD	11	40 mg	Coadministration on day 8	11.7	0.24	0.67	0.56
CYP2C9								
Fluconazole (moderate inhibitor)	200 mg QD	10	40 mg	Coadministration on day 7	24.9	0.79	1.42	1.80
fluvoxamine (mild inhibitor)	50 (36.65) mg QD^c^	10	40 mg	Coadministration on day 7	18.4	0.48	1.05	1.08
Substrates	Dose	Treatments	HQP1351 dose	HQP1351 treatments (days)	C_max_inh_ (ug/mL)	AUC_360___inh_ (h•ug/L)	C_max_ ratio	AUC ratio
CYP2C9								
Tolbutamide	500 mg	Coadministration on day 13	40 mg QOD	27	44.0	0.82	0.92	0.71
(S)-Warfarin	10 mg	Coadministration on day 13	40 mg QOD	27	1.20	0.06	0.98	0.72
CYP2C19								
Omeprazole	20 mg	Coadministration on day 13	40 mg QOD	27	0.32	0.002	1.01	1.02
CYP1A2								
Caffeine	150 mg	Coadministration on day 13	40 mg QOD	27	3.99	0.03	0.93	0.69
CYP2B6								
Bupropion	150 (130.2) mg^d^	Coadministration on day 13	40 mg QOD	27	0.104	0.002	0.95	0.91
P-gp								
Dabigatran etexilate	150 mg	Coadministration on day 13	40 mg QOD	27	2.63	14.4	1.00	1.00
Digoxin	0.5 mg	Coadministration on day 13	40 mg QOD	27	1.77	28.3	1.00	1.00

Cmax_inh: Maximum plasma substrates concentration following coadministration with modulators; AUC_96__inh: Area under the plasma substrates concentration curve from 0 to 96 h following coadministration with modulators; AUC_360__inh: Area under the plasma concentration curve from 0 to 360 h following coadministration with modulators.

^a^ Rifampicin is also a moderate inducer of CYP2C9.

^b^ Phenobarbital is the Simcyp default drug model, which also induced CYP2C9.

^c^ Fluvoxamine maleate dose is 50 mg, which contains fluvoxamine 36.65 mg.

^d^ Bupropion hydrochloride sustained-release tablets dose is 150 mg, which contains fluvoxamine 130.2 mg. BID, two times a day; QD, every day; QOD, every other day; TID, three times a day.

To evaluate the effect of liver function on olverembatinib pharmacokinetics, we performed the simulation in virtual patients with liver function injury classified as mild (A), moderate (B) and severe (C) by the Child-Pugh (CP) classification, including a prespecified dose scheme (Table 3. Ten virtual trials were simulated for each scenario to assess interstudy variability, and 10 subjects (aged 20–50 years and 50% female) participated in each simulated trial. All DDI model simulations were conducted with the virtual “Sim-Chinese healthy volunteers” population except for a P-gp-mediated DDI simulation, which was conducted with the representative population “Sim-Chinese healthy volunteers” population. All liver cirrhosis simulations were conducted with the default virtual population “Sim-Healthy volunteers, “Sim-Cirrhosis CP-A, “Sim-Cirrhosis CP-B,” and “Sim-Cirrhosis CP-C″ populations. Except for olverembatinib, all compound files in Simcyp V.19 default were used.

**TABLE 3:** Simulated C_max_ and AUC_0-96_ of olverembatinib (HQP1351) in different liver cirrhosis populations.

Population	Description	HQP1351 dose	C_max_ (ng/ml)	AUC_0-96_ (h•ng/mL)	C_max_ ratio	AUC_0-96_ ratio
Healthy Caucasian population	Caucasian healthy population	40 mg SD	15.2	292	NA	NA
Sim-cirrhosis CP-A	Caucasian mild cirrhosis population	40 mg SD	17.6	356	1.16	1.22
Sim-cirrhosis CP-B	Caucasian moderate cirrhosis population	40 mg SD	24.2	522	1.59	1.79
Sim-cirrhosis CP-C	Caucasian severe cirrhosis population	40 mg SD	28.4	621	1.87	2.13

C_max_ ratio: The ratio of the Cmax in the liver cirrhosis population and Cmax in Caucasian healthy population; AUC, ratio: The ratio of the area under the curve (AUC) in the liver cirrhosis population and AUC, in Caucasian healthy population; CP, Child-Pugh; NA, not applicable; SD, single dose.

## 3 Results

### 3.1 Model construction

**FIGURE 2:**
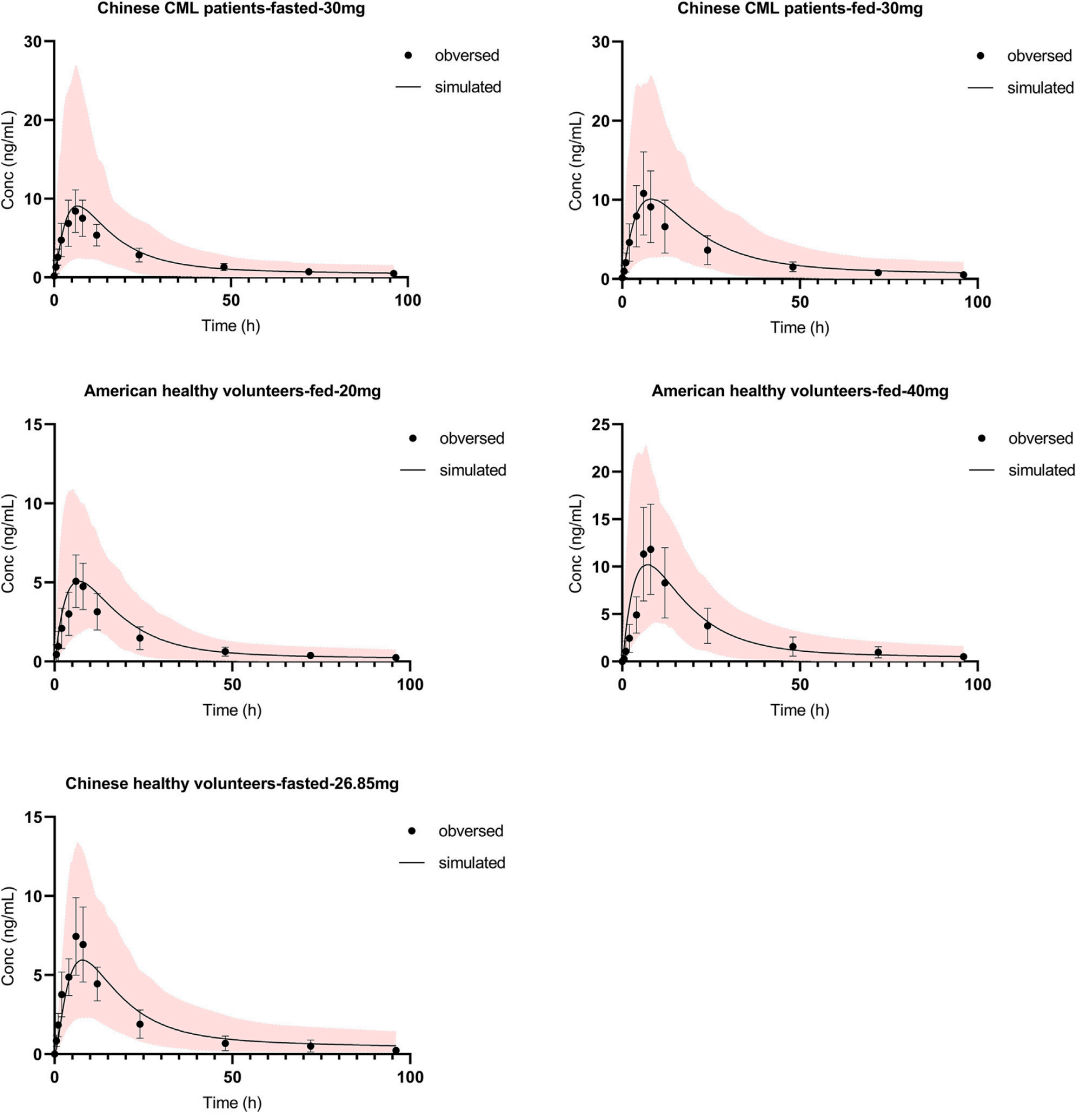
Olverembatinib PBPK model verification. Observed (mean and standard deviation, dots and bar) and simulated (solid lines) mean plasma concentration-time profiles of olverembatinib in different populations following single oral doses of olverembatinib (30, 20, 40 or 26.85 mg). The shaded area is the 5–95% range in concentrations from 1,000 simulated healthy Chinese or American adult subjects.

Absorption and distribution behaviors of olverembatinib were effectively predicted using first-order absorption kinetics with a minimal PBPK model. Using the same dose regimen as in dosed patients, a simulation was performed. The predicted plasma concentration profiles after single oral 30 mg doses (fasting or fed) were consistent with observed profiles in Chinese patients with CML in the food effect study (Figure 2). C_max_ ratios (predicted/observed) in fasting and fed conditions were 1.13 and 1.02, respectively. AUC ratios (predicted/observed) in fasting and fed conditions were 0.95 and 1.12, respectively. Similarly, predicted concentration profiles after a single oral dose of 20 or 40 mg were consistent with those observed data in healthy American volunteers. The C_max_ and AUC ratio (predicted/observed) ranges were 0.88–1.06 and 0.88 to 1.03, respectively. In the mass balance study, the observed olverembatinib concentration in healthy Chinese volunteers were within the prediction interval. The C_max_ and AUC ratios (predicted/observed) were 0.76 and 1.12, respectively.

### 3.2 Model validation

**FIGURE 3:**
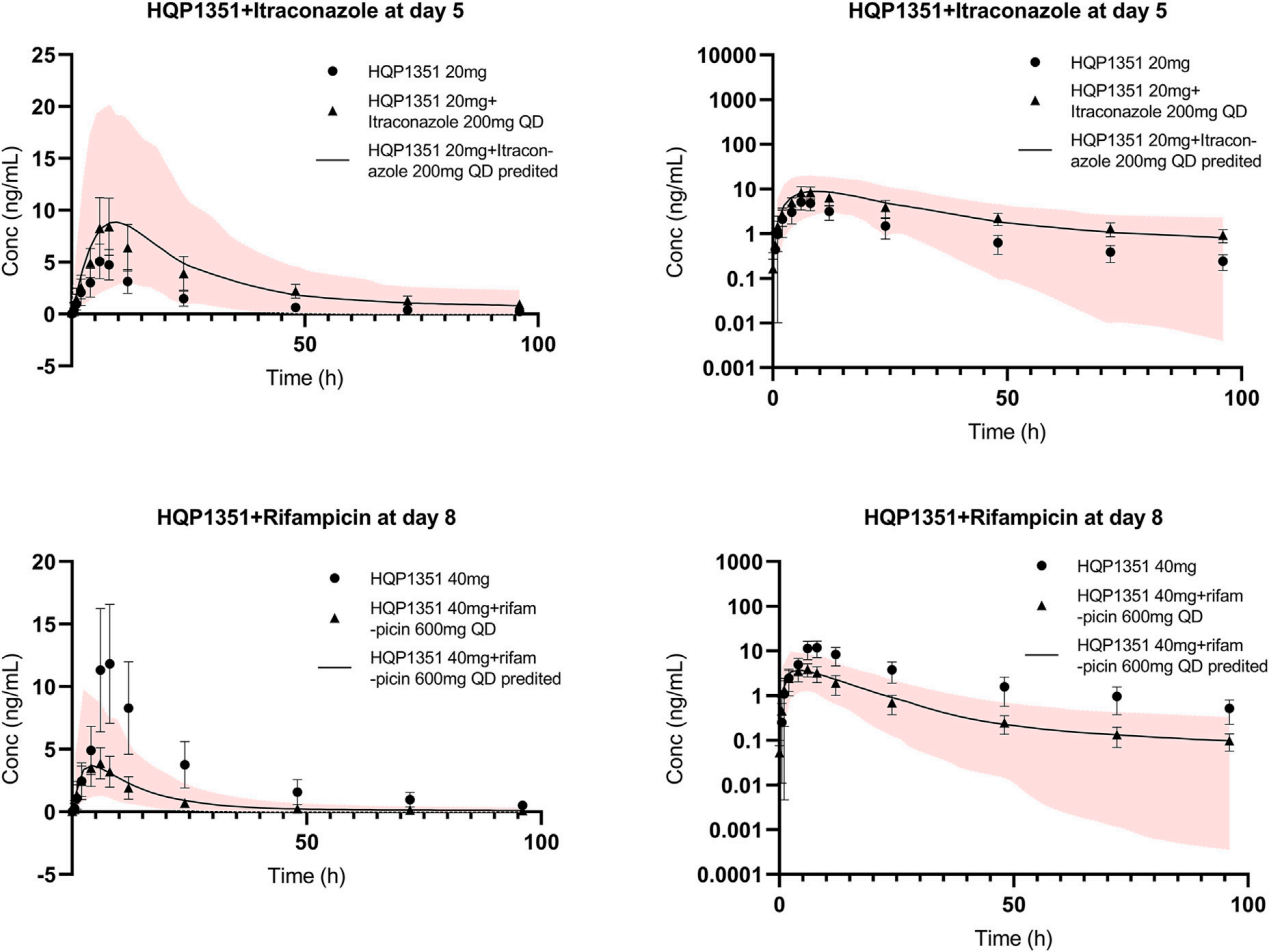
Further verification of olverembatinib PBPK model. Observed (mean and standard deviation, dots for single dose, triangle for coadministration and bar) and simulated (solid lines for coadministration) mean plasma concentration-time profiles of olverembatinib in healthy American volunteers following oral doses of olverembatinib (left panels = linear scale; right panels = semi-log scale). The shaded area is the 5%–95% range in concentrations from 1,000 simulated healthy American adult subjects.

The applicability of the PBPK model to assess the magnitude of drug interactions between olverembatinib and CYP3A4 perpetrators was verified. The model simulated the concentration-time profiles of 20 mg of olverembatinib administered with CYP3A4 inhibitor itraconazole in the clinical DDI study (Figure 3). Itraconazole’s effect on olverembatinib was simulated in profiles for changes in C_max_ and AUC (Supplementary Table S3). The observed C_max_ and AUC ratios of olverembatinib in the presence or absence of itraconazole were 1.74 and 2.63, respectively. The corresponding PBPK model predicted C _max_ and AUC ratios of 1.69 and 2.22, respectively, which were consistent with observed values in the clinical DDI study.

### 3.3 Model simulation

The effect of CYP3A4 inducer rifampicin on olverembatinib was demonstrated by simulating the pharmacokinetic profiles of the TKI when coadministered with the antibiotic. The simulation was acceptable when compared with the observed concentration-time profiles of olverembatinib among healthy American volunteers in the rifampicin DDI study (Figure 3). Observed C_max_ and AUC ratios of olverembatinib in the presence and absence of rifampicin were 0.36 and 0.24, respectively, compared to predicted ratios of 0.38 and 0.27, respectively. The PBPK model-predicted mean C_max_ and AUC ratios of olverembatinib (at 40 mg) with or without rifampicin were less than 1.5 times the observed values (Supplementary Table S3).

#### 3.3.1 Simulation of olverembatinib as a DDI victim

The constructed PBPK model was used to simulate other untested clinical DDI scenarios for olverembatinib during coadministration with CYP3A4 and CYP2C9 inhibitors and inducers in healthy Chinese volunteers. Simulated olverembatinib pharmacokinetic parameters and corresponding ratios with and without coadministration of CYP3A4 and CYP2C9 modulators are outlined (Table 2).

PBPK simulations indicated that olverembatinib AUC_0–96h_ (area under the plasma drug concentration-time curve from 0 to 96 h) may increase by approximately 2.39- and by 1.80- to 2.39-fold during coadministration with strong and moderate CYP3A4 inhibitors, respectively. Simulations suggested that the strong and moderate CYP3A4 inducers may decrease the AUC_0–96h_ of olverembatinib by 0.29- and 0.56- to 0.35-fold, respectively. Based on the PBPK model simulations, predicted ratios of olverembatinib AUC_0–96h_ in the presence or absence of moderate and mild CYP2C9 inhibitors were 1.80 and 1.08, respectively.

#### 3.3.2 Simulation of olverembatinib as a DDI perpetrator

To assess the potential effects of olverembatinib on the pharmacokinetics of CYP2C9, CYP2C19, CYP1A2, CYP2B6, and CYP3A4 substrate drugs, the predicted pharmacokinetic parameters and mean C_max_ and AUC ratios of substrate drugs with or without olverembatinib were simulated (Table 2).

Tolbutamide (which interferes with binding of anticoagulant warfarin enantiomers) and (S)-warfarin are substrates of CYP2C9. PBPK simulations indicate that tolbutamide and (S)-warfarin AUC_0–360h_ (area under the plasma drug concentration-time curve from 0 to 360 h) may decrease by approximately 0.71- and 0.72-fold, respectively during coadministration with olverembatinib. Similarly, simulations suggested that olverembatinib may increase the AUC_0–360h_ of CYP2C19 substrate and proton pump inhibitor omeprazole by 1.02-fold and decrease the AUC_0–360h_ of CYP1A2 substrate of caffeine (anaesthetic) and CYP2B6 substrate and antidepressant bupropion by 0.69- and 0.91-fold, respectively.

As a DDI perpetrator, olverembatinib has no or limited impact on the enzyme activity of CYP2C9, CYP2C19, CYP1A2, or CYP2B6. Finally, we assessed the potential effects of olverembatinib on the pharmacokinetics of P-gp substrates dabigatran etexilate (low-molecular-weight prodrug of the direct thrombin inhibitor) and the cardiac glycoside digoxin. The AUC_0–360h_ ratio for dabigatran etexilate or digoxin in the presence or absence of olverembatinib was 1.0 each.

#### 3.3.3 Simulation of olverembatinib in liver cirrhosis populations

In this simulation, we used a liver cirrhosis population model based on Caucasian people. The ratio of the AUC in liver cirrhosis and healthy populations was assumed to be equal in both Caucasian and Chinese people. PBPK simulations indicated that predicted ratios of olverembatinib AUC_0–96h_ among patients in cirrhosis CP-A, CP-B, and CP-C, relative to healthy volunteers, were 1.22, 1.79, and 2.13, respectively. For C_max_, the model-predicted ratios in patients with CP-A, CP-B, and CP-C cirrhosis, relative to healthy volunteers, were 1.16, 1.59, and 1.87, respectively (Table 3).

## 4 Discussion

This study developed a model-based approach for comprehensive evaluation of olverembatinib DDI profiles, including evaluation of its potential as a victim of CYP3A4 and CYP2C9 and as a perpetrator for CYP2C9, CYP2C19, CYP1A2, CYP2B6, and P-gp substrates. PBPK modeling was used to assess metabolic DDI risks as part of the clinical pharmacology strategy for the development of olverembatinib. Robustness of the current PBPK model was demonstrated by the similarity of model predictions with observed clinical data after a single dose of olverembatinib across various drug interaction pathways. Because hepatic metabolism is the principal pathway governing elimination of olverembatinib, the effect of liver function injury on exposure to olverembatinib was evaluated by simulating pharmacokinetic profiles in patients with liver cirrhosis.

Olverembatinib is metabolized by multiple CYP isozymes, with higher relative contributions with CYP3A4 and CYP2C9 (60.0% and 21.6%; data from human liver microsome study). The clinical DDI study indicated that the AUC of olverembatinib (20 or 40 mg single dose) increased 2.61-fold (or by 163%) after itraconazole treatment (200 mg daily [QD]) and decreased 0.24-fold (or by 76%) after rifampicin administration. In addition, a PBPK modeling simulation demonstrated that a strong CYP3A4 inhibitor increased olverembatinib AUC by 139%. Moderate and mild CYP3A4 inhibitors (some of which overlap as CYP2C9 inhibitors) increased olverembatinib exposures by 80%–139% and 8%, respectively.

Strong and moderate CYP3A4 inducers (some of which overlap as CYP2C9 inducers) decreased olverembatinib exposure by 44%–71%. In our study, moderate CYP3A4 inhibitor and antibiotic erythromycin was administered at a higher-than-routine dose (500 mg every 6 h) and hence showed an inhibitory effect similar to that with strong CYP3A4 inhibitor itraconazole. After adjustment of the erythromycin dose to 500 mg 3 times a day, the mean C_max_ and AUC_0-t_ ratios for olverembatinib in the presence or absence of erythrocin were 1.60 and 2.39, respectively. Both CYP3A4 and CYP2C9 were inhibited by fluconazole and antidepressant fluvoxamine and induced by rifampicin and the barbiturate phenobarbital. Olverembatinib exposures changed significantly after coadministration with these drugs. Based on the clinical DDI study and predictions of the PBPK model, coadministration of olverembatinib with strong and moderate CYP3A4 inhibitors or inducers is not recommended.

*In vitro* studies suggested that olverembatinib is a weak CYP2C9 and CYP2C19 inhibitor, and a weak CYP1A2, CYP2B6 and CYP2C9 inducer. The PBPK model was used to evaluate the DDI potential of olverembatinib and CYP/P-gp substrates. Olverembatinib decreased tolbutamide and (S)-warfarin (CYP2C9 substrates), caffeine (CYP1A2 substrate), and bupropion (CYP2B6 substrate) exposures by 28%–29%, 31%, and 9%, respectively. Olverembatinib increased omeprazole (CYP2C19 substrate) exposure by 2% but did not change exposures of dabigatran etexilate and digoxin (P-gp substrates). As a DDI perpetrator, olverembatinib had no or limited impact on activities of CYP1A2, CYP2B6, CYP2C9, CYP2C19, and P-gp.

In addition, olverembatinib is neither a substrate nor inhibitor of OATP1B1 (organic anion transporting polypeptide 1B1), OATP1B3 (organic anion transporting polypeptide 1B3), OAT1 (organic anion transporter 1), OAT3 (organic anion transporter 3), OCT2 (organic cation transporter 2), MATE1 (multidrug and toxin extrusion 1), or MATE2K (multidrug and toxin extrusion 2). Olverembatinib also showed no obvious *in vivo* inhibition on P-gp and BCRP (breast cancer resistance protein) and exerted a negligible perpetrator effect on substrates of common CYP enzymes and transporters. However, these findings will need to be further validated by additional clinical studies.

Hepatic elimination is an important mechanism that regulates overall clearance of olverembatinib. In the current simulation, we show that respective exposures of olverembatinib in Caucasian populations with mild, moderate, and severe cirrhosis were 22%, 79%, and 113% higher than in healthy volunteers. Because no clinical data were obtained before this study, the olverembatinib pharmacokinetic assessment in liver function injury populations was not validated. The simulation results indicate that patients with mild liver injury can receive the same dose of olverembatinib as those with normal liver function. An appropriate dose adjustment should be considered for patients with moderate liver injury by weighing the benefits and risks. However, in patients with severe cirrhosis, olverembatinib is not recommended. Additional clinical studies in patients with liver cirrhosis are needed to optimize the dose regimen.

A limitation of this study is that the transporters mediated disposition was not integrated in PBPK model since olverembatinib is a substrate of P-gp, BCRP (breast cancer resistance protein). The effects of transporters expressed on the gut and liver have not been investigated.

Besides, the B/P ratio was obtained from healthy volunteer blood, however, in patients with CML, too many myeloid cells become granulocytes (red blood cells, platelets and several white blood cell types). So, red blood cell count might change B/P ratio in CML patients. The B/P ratio of olverembatinib in CML patients need a further study. Furthermore, the model simulation for liver cirrhosis patients was not validated by clinical data. We believe that further research is needed on this point.

## 5 Conclusion

A robust PBPK model of olverembatinib was constructed and validated to predict CYP3A4-and CYP2C9-mediated drug interactions. The validated PBPK model was utilized to forecast DDI risks where no clinical data were available. Simulations suggested that no dose adjustment for olverembatinib is required when the TKI is coadministered with mild CYP3A4 and CYP2C9 inhibitors. With moderate and strong CYP3A4 or CYP2C9 inhibitors and inducers, coadministration is not recommended. Dose adjustment might be not needed in light liver cirrhosis patients with CP-A. Moderated liver cirrhosis patients should use olverembatinib with caution and are suggested to monitor liver function. But the severe liver cirrhosis patients are not suggested to use olverembatinib.

## Data Availability

The original contributions presented in the study are included in the article/Supplementary Material, further inquiries can be directed to the corresponding authors.
