# Supplementary material for: Potential drug-drug interaction of olverembatinib (HQP1351) using physiologically based pharmacokinetic models
Source: Front Pharmacol. 2022 Dec 13;13:1065130. doi: 10.3389/fphar.2022.1065130 (PMC9792776; doi:10.3389/fphar.2022.1065130)
Supplement: Supplementary file 1 [file DataSheet1.docx]

# Supplementary Information

# Olverembatinib clinical study data used in physiologically based pharmaco-kinetic study: Clinical study information are summarized in Supplementary Table S1, which included a food effect study, human mass balance study, and clinical drug-drug interaction study with a strong CYP3A inhibitor/inducer.

# Olverembatinib physiologically based pharmacokinetic model verification: Observed and Simcyp-simulated pharmacokinetic parameters of olverembatinib following a single dose and coadministration with itraconazole and rifampicin are summarized in Supplementary Tables S2 and S3.

**Olverembatinib preclinical study data used in physiologically based pharmaco-kinetic study:** The detailed results of some preclinical parameters: fu, B/P ratio, CLint_CYP_, Ind_maxCYP_ and IndC_50CYP_ are summarized in Supplementary Table S4, S5, S6 and S7

**Supplementary Table S1**
Olverembatinib clinical study data used in physiologically based pharmacokinetic study.

| **Clinical study** | **Population** | **Study description** | **Dose regimen** |
| --- | --- | --- | --- |
| NCT03882281 | Chinese patients with CML  (n = 12) | Food effect study | 30 mg SD fasting or 30 mg SD fed  (30 minutes after eating) |
| NCT04126707 | Chinese healthy volunteers  (n = 6) | Human mass balance study | 100 μCi/30 mg suspension SD fasting  (mean dose = 26.85 mg) |
| NA | White healthy volunteers  (n = 32) | Clinical DDI study with strong CYP3A inhibitor/inducer | 20 mg SD (DDI with itraconazole,  200 mg QD)/40 mg SD (DDI with rifampin, 600 mg QD) |

CML, chronic myeloid leukemia; DDI, drug-drug interaction; NA, not registered on clinicaltrials.gov; QD, once daily; SD, single dose.

**Supplementary Table S2**
Observed and Simcyp-simulated pharmacokinetic parameters of olverembatinib in Chinese patients with chronic myeloid leukemia (CML), Chinese healthy volunteers, or White healthy volunteers after a single dose.

| **Trial** | | | | **C_max_ (ng/mL)** | | | **T_max_(h)*** | | | **AUC (h🞄ng/mL)** | | |  |
| --- | --- | --- | --- | --- | --- | --- | --- | --- | --- | --- | --- | --- | --- |
| **Population** | **Fed/fasting** | **Dose (mg)** | **Dose regimen** | **Obs.** | **Pred.** | **Ratio (P/O)** | **Obs.** | **Pred.** | **Ratio (P/O)** | **Obs.** | **Pred.** | **Ratio (P/O)** | |
| Chinese CML patients | Fasting | 30 | SD | 8.72 | 9.88 | 1.13 | 6.00 | 8.31 | 1.39 | 237 | 226 | 0.95 | |
| Chinese CML patients | Fed | 30 | SD | 10.8 | 11.0 | 1.02 | 6.00 | 10.2 | 1.69 | 276 | 308 | 1.12 | |
| White healthy volunteers | Fed | 20 | SD | 5.26 | 5.59 | 1.06 | 8.00 | 9.02 | 1.13 | 125 | 129 | 1.03 | |
| White healthy volunteers | Fed | 40 | SD | 12.7 | 11.2 | 0.88 | 8.00 | 9.17 | 1.15 | 302 | 265 | 0.88 | |
| Chinese healthy volunteers | Fasting | 26.85 | SD | 8.09 | 6.17 | 0.76 | 6.00 | 8.36 | 1.39 | 153 | 171 | 1.12 | |
| Mean ratio | | | |  |  | **0.97** |  |  | **1.35** |  |  | **1.02** |  |

^a^The food effect study included 12 patients with CML. Because pharmacokinetic concentration data of 3 patients were obviously abnormal, these concentrations were excluded. *Median value. AUC, area under the drug concentration time curve; C_max_, maximum concentration; T_max_, time to reach C_max_; Obs., observed pharmacokinetic parameters from clinical trials; Pred., predicted pharmacokinetic parameters from model simulation; P/O, ratio of predicted to observed pharmacokinetic parameters.

**Supplementary Table S3**
Observed and Simcyp-simulated pharmacokinetic parameters of olverembatinib in healthy White volunteers after coadministration with CYP3A4 substrates itraconazole and rifampicin.

| **Scenario** | **Parameters** | **Obs.** | **Pred.** | **Ratio (P/O)** |
| --- | --- | --- | --- | --- |
| Itraconazole (Inhibitor) | C_max_ ratio | 1.74 | 1.69 | 0.97 |
|  | AUC ratio | 2.63 | 2.22 | 0.84 |
|  | C_max_ (ng/mL) | 9.14 | 9.54 | 1.04 |
|  | T_max_ (h)* | 6.00 | 11.5 | 1.92 |
|  | AUC (h🞄ng/mL) | 330 | 293 | 0.89 |
| Rifampicin (Inducer) | C_max_ ratio | 0.36 | 0.38 | 1.06 |
|  | AUC ratio | 0.24 | 0.27 | 1.10 |
|  | C_max_ (ng/mL) | 4.54 | 4.28 | 0.94 |
|  | T_max_ (h)* | 6.00 | 5.86 | 0.98 |
|  | AUC (h🞄ng/mL) | 73.0 | 70.7 | 0.97 |

C_max_ ratio, mean ratio (in the presence/absence of itraconazole or rifampicin) of the C_max_ for olverembatinib; AUC ratio, mean ratio (in the presence/absence of itraconazole or rifampicin) of the AUC for olverembatinib. *Median value; AUC, area under the drug concentration time curve; C_max_, maximum concentration; T_max_, time to reach C_max_; Obs., observed pharmacokinetic parameters from clinical trials; Pred., predicted pharmacokinetic parameters from model simulation; P/O, ratio of predicted to observed pharmacokinetic parameters.

**Supplementary Table S4**

Fu of olverembatinib with three test concentration in human plasma

| species | drug | test concentration | fu (N=3) | mean fu |
| --- | --- | --- | --- | --- |
| human | olverembatinib | 80 ng/ml | 0.0007 | 0.0005 |
|  |  | 400 ng/ml | 0.0004 |  |
|  |  | 4000 ng/ml | 0.0004 |  |

**Supplementary Table S5**

B/P ratio of olverembatinib in healthy volunteers

| species | drug | sample | mean conc (N=3) | sample | mean conc (N=3) | B/P ratio | mean B/P ratio |
| --- | --- | --- | --- | --- | --- | --- | --- |
| human | olverembatinib | blood L | 0.395 | plasma L | 0.25 | 1.58 | 1.29 |
|  |  | blood M | 4.01 | plasma M | 2.95 | 1.36 |  |
|  |  | blood H | 43.5 | plasma H | 45.9 | 0.95 |  |

L, low concentration; M, median concentration; H, high concentration;

**Supplementary Table S6**

Intrinsic clearance of olverembatinib and positive control drug in human recombinant enzymes CYP2C9 and CYP3A4

| CYP | Drug | Cl_int_ (µL/min/pmol rCYP) | CYP Abundance (pmol/mg protein) | Relative contri-bution (%) |
| --- | --- | --- | --- | --- |
| CYP2C9 | Diclofenac | 2.965 | 96 | NA |
| CYP3A4 | Midazolam | 3.083 | 108 | NA |
| CYP2C9 | olverembatinib | 0.022 | 96 | 8.9 |
| CYP3A4 |  | 0.2 | 108 | 91.1 |

Diclofenac and midazolam are positive control drug of CYP2C9 and CYP3A4, respectively.

**Supplementary Table S7**

Fold Induction, EC50, and Emax of CYP mRNA by olverembatinib in Culture Human Hepatocytes

| Test Compound | Human hepatocytes | Isoform | Concentrations (µM)/mRNA Fold Induction | | | | | | EC_50_ (µM) | Mean EC_50_ | E_max_ (Fold Induction) | Mean  E_max_ |
| --- | --- | --- | --- | --- | --- | --- | --- | --- | --- | --- | --- | --- |
|  |  |  | 0.0033 | 0.01 | 0.033 | 0.1 | 0.33 | 1 |  |  |  |  |
| olverembatinib | HUM182851 | CYP1A2 | 1.00 | 1.50 | 1.84 | 2.31 | 3.31 | 6.07 | 0.150 | 0.15 | 6.26 | 6.26 |
|  | AIH | CYP2B6 | 1.29 | 1.18 | 2.19 | 4.30 | 5.29 | 13.4 | 0.828 | 0.559 | 23.9 | 17.9 |
|  | HUM182851 |  | 1.12 | 1.57 | 2.27 | 3.89 | 4.67 | 9.95 | 0.290 |  | 11.9 |  |
|  | AIH | CYP2C9 | 1.08 | 0.878 | 1.73 | 2.79 | 5.33 | 10.6 | 0.584 | 0.454 | 16.5 | 17.15 |
|  | HUM182851 |  | 1.18 | 1.49 | 2.57 | 4.47 | 8.04 | 13.8 | 0.323 |  | 17.8 |  |

AIH and HUM182851 are different lot human hepatocytes.
